# Anal Sphincter Reconstruction Using the Posterior Sagittal Approach for Pediatric Perineal Trauma

**DOI:** 10.1055/a-2487-5249

**Published:** 2024-12-19

**Authors:** Julia Ann Ryan, Thomas O. Xu, Christina Ho, Briony K. Varda, Veronica Gomez-Lobo, Allison Mayhew, Erin Teeple, Andrea Badillo, Christina Feng, Marc A. Levitt

**Affiliations:** 1 Department of Colorectal and Pelvic Reconstruction, Children's National Hospital, Washington, District of Columbia, United States; 2 School of Medicine and Health Sciences, The George Washington University, Washington, District of Columbia, United States; 3 Department of Surgery, Colorectal and Pelvic Reconstructive Surgery, Children's National Hospital, Washington, District of Columbia, United States; 4 Department of Urology, Children's National Medical Center, Washington, District of Columbia, United States; 5 Department of Urology, Children's National Hospital, Washington, District of Columbia, United States; 6 Division of Pediatric and Adolescent Gynecology, Children's National Hospital, Washington, District of Columbia, United States; 7 Department of Pediatric and Adolescent Gynecology, National Institute of Child Health and Human Development, Rockville, Maryland, United States; 8 Department of Pediatric and Adolescent Gynecology, Children's National Medical Center, Washington, District of Columbia, United States; 9 Department of Colorectal and Pelvic Reconstruction, Children's National Medical Center, Washington, District of Columbia, United States

**Keywords:** perineal trauma, sphincter reconstruction, posterior sagittal approach

## Abstract

Traumatic perineal injuries are rare but can result in significant morbidity, particularly when the anal sphincter is injured. The management of such injuries in the pediatric population is rarely noted in the literature. We aimed to describe reconstruction in such patients using lessons learned in reoperative anorectal malformation surgery. This is a single-institution retrospective case series of three pediatric patients who were referred to our institution with pelvic trauma who underwent anal sphincter reconstruction. Three patients aged 5 (female), 12 (male), and 13 (female) years were referred for reconstruction following pelvic trauma involving the anal sphincter, perineal body, and genitourinary system. All three underwent multidisciplinary evaluation with urology and gynecology (for females) and a subsequent repair with anal sphincter reconstruction utilizing a posterior sagittal approach. Two patients had ostomy reversal with appendicostomy for antegrade continence enemas and regained voluntary fecal control. The third patient is awaiting colostomy reversal but has regained volitional urinary control after urethral reconstruction. The experience gained from using the posterior sagittal anorectoplasty approach in reoperations for patients with anorectal malformations can be applied to cases of rectal trauma. Key aspects include mobilizing the rectum, repairing the sphincters, and placing them around the anus, and in females, reconstructing the perineal body. Pediatric pelvic trauma can cause devastating disruptions of physiology and are difficult to treat. Experience from reoperations for anorectal malformations can be applied to these cases, including the use of a multidisciplinary team and posterior sagittal approach.

## Introduction

Perineal trauma, while rare, is a devastating injury in the pediatric population, representing 0.2 to 8% of all pediatric trauma cases. 1 2 These injuries result from various mechanisms, including blunt and penetrating trauma, with falls and motor vehicle collisions the most common, and sexual abuse as an additional mechanism. 3 4 The trauma often causes compression of the perineum against the bony pelvis, leading to a range of injury patterns, with one of the most severe being complete disruption of the anal sphincter complex. 5 Initial management typically includes diversion with a colostomy and stabilization of other associated injuries, often involving the genitourinary system. Wound management factors into most cases. After initial recovery, the long-term management of these anorectal injuries is difficult, especially when involving the anal sphincter complex, with the goal to restore fecal continence. There is limited literature on reconstruction following such injuries, especially in the pediatric population.

This study presents three cases of pediatric patients who sustained extensive pelvic trauma and were initially stabilized at outside institutions. They were referred to our colorectal and pelvic reconstruction center and had collaborative visits with pediatric colorectal surgery, urology, gynecology, orthopaedics, and plastic surgery.

## Case Reports

### Case 1

A 12-year-old female sustained extensive pelvic trauma from an industrial machinery accident resulting in injuries to the rectum, vagina, and bladder with an open book pelvic fracture and a right femur fracture in addition to a Morel–Lavallée lesion, a closed degloving soft tissue injury, of the right groin. She underwent exploratory laparotomy with small bowel resection and partial colectomy due to devitalized bowel and an end sigmoid colostomy. Her perineal laceration and bladder perforation were repaired, and she also underwent a vaginoplasty, perineoplasty, and anoplasty. Given the extent of her degloving injury with associated fractures, the patient had multiple operations with the orthopaedics and plastics teams at an outside hospital, including use of split thickness skin graft involving 4% of her total body surface area in the region of the injury, in addition to a right quadricepsplasty and right femur osteotomy.

Four months after her initial injury and stabilization, the patient had a cystourethroscopy and vaginoscopy that demonstrated a normal bladder and no signs of a rectovesical fistula. Her exam at that time was notable for an absent perineal body and focal areas of poor sphincter contraction on “sphincter mapping.” The patient was also noted to have a gaping vaginal introitus, and the cervix could be visualized without speculum insertion into the vaginal canal. She was referred to our institution for evaluation.

At the time of her presentation to our institution, the patient was a year and a half out from her initial trauma. She was stooling through her end sigmoid colostomy and was urinating normally. A distal colostogram and contrast enema demonstrated no stricture or fistulae. A computed tomography scan of the pelvis revealed an osteotomy fragment just lateral to the right pubic symphysis. We performed an exam under anesthesia (EUA) and vaginoscopy to assess her anatomy prior to planning a reconstruction and found significant laxity of the levator muscles bilaterally and an enlarged introital opening. The perineal body was short with significant surrounding scar tissue and there was lateral displacement of the urethral meatus secondary to the bony spur protruding from the pubic symphysis.

Anal evaluation demonstrated an intact anal sphincter from the 1 to 9 o'clock position with a patent anus without stricture or prolapse. Given these findings, we performed a perineal body sphincter reconstruction, posterior colporrhaphy, perineoplasty, introitoplasty, and labiaplasty with removal of the pelvic bone spur.

A posterior sagittal approach was used for the dissection of the perineal structures. A dissection of the posterior edge of the vaginal mucosa and the perineal body skin was performed, developing a plane between the anterior rectum and posterior vagina. An incision was then made on the vaginal mucosa to expose the sphincter muscles, which were then plicated in the midline, anterior to the anus. This approach was similar to the perineal body reconstruction required for reoperations in patients with anorectal malformations who have suffered from perineal body dehiscence. 6 7 The rectovaginal space, vaginal mucosa, bulbocavernosus muscles, and skin of the perineal body were then reapproximated. The vaginal mucosa between the clitoral hood and urethral meatus was incised to visualize the bone spur, which was removed, and the vaginal mucosa then reapproximated. At the end of the case, a digital rectal exam with an electrical stimulator was performed, which showed a concentric ring of muscle contraction at the reconstructed sphincter complex.

The patient had an uneventful postoperative course and was discharged home on day 1. After complete healing, an EUA showed her to have a well-healed perineum with an adequate perineal body. The anus was well healed without stricture. The colostomy was taken down and a neo-Malone appendicostomy was performed (the patient had a prior appendectomy). The appendicostomy was placed to offer antegrade cleaning of the colon to be utilized as a bridge to achieving continence by practicing the use of the reconstructed sphincter muscles to hold onto and release the flush. 8

One month after discharge, the patient was seen for follow-up, stooling with assistance of the antegrade continence enema and without stool accidents. A year and a half after her reconstructive surgery, the patient was completely continent of stool and was no longer using Malone. She was stooling spontaneously one-to-two times per day, without pain or accidents. The Malone site was allowed to spontaneously close.

### Case 2

A 13-year-old male sustained a transection of the anus and prostatic urethra with extensive pelvic and lower extremity fractures after being struck by a car while riding his bicycle. He underwent exploratory laparotomy, sigmoid colostomy, cystostomy with suprapubic catheter placement, and multiple fracture repairs. Most of the patient's soft tissue destruction was to the left of his anus, and his rectum was completely avulsed off of the anoderm. His pelvic floor muscles were partially transected, although on stimulation, the patient was shown to have adequate contraction of the right sphincter complex with minimal contraction of the left and posterior aspect. He then presented to our institution for reconstructive surgical evaluation.

**Fig. 1:**
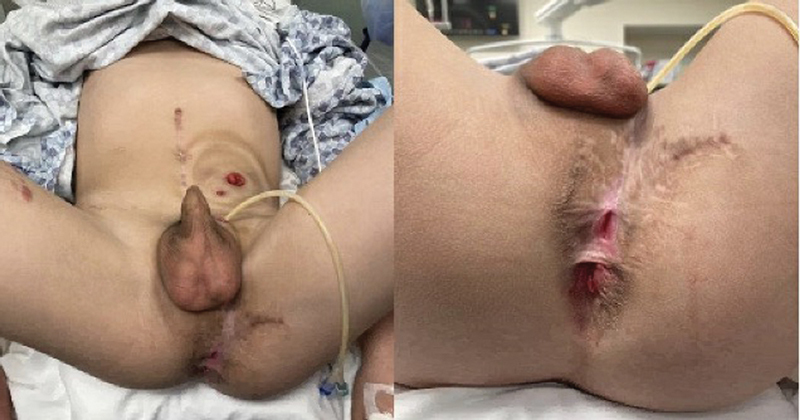
Findings from exam under anesthesia with significant scarring to the left anterior aspect of the anus and perineum.

To assess his anatomy, the patient underwent an EUA with cystoscopy, retrograde urethrogram, cystogram, and distal colostogram with the colorectal and urology teams. He was noted to have significant scarring to the left anterior aspect of the anus and perineum ( Fig. 1 ).

There was anoderm present but no rectal lumen; however, good sphincter contraction was appreciated mostly on the right side of the anal opening. On distal colostogram, the rectum ended blindly just below the pubococcygeal line, 4.4 cm from the anal opening. On retrograde urethrogram and cystoscopy, the patient was found to have a 2 cm stricture of his prostatic/membranous urethra, just distal to the external urethral sphincter. Measurement from the urethral meatus to the stricture was 11.8 cm, and from the bladder neck to the stricture was 1.9 cm.

The patient's reconstruction was planned in two stages. He first underwent posterior sagittal anorectoplasty (PSARP) with anal sphincter reconstruction. The lumen of the native rectum was identified and circumferentially mobilized to the level of the peritoneal reflection to ensure adequate length to the anal opening. The mobilized neorectum was placed in the center of the isolated anoderm, and a colo-anal anastomosis was performed. The external sphincter, noted to be off midline toward the patient's right, was then split in the middle and tacked circumferentially to the rectal pull through.

He then underwent a robotic-assisted laparoscopic excision and primary anastomosis urethroplasty with perineal exposure at another institution with vast experience with primary robotic urethral repairs. One month after this repair, the patient was able to volitionally void with only mild stress urinary incontinence but was otherwise dry for urine. A cystoscopy demonstrated a well-healed urethroplasty and anorectoplasty. On exam, he now has an excellent circumferential squeeze around the anus on digital exam. His colostomy closure with Malone appendicostomy as a bridge to continence is planned.

### Case 3

A 5-year-old female sustained extensive pelvic trauma including injuries to the rectum, vagina, and bladder with associated transection of the left femoral artery and vein, pelvic fracture, and soft tissue injury of the lower abdominal wall after being struck by a vehicle as a pedestrian. Her mother held pressure on the femoral artery until emergency medical services arrived. She underwent emergent repair of her left femoral vessels with creation of a transverse loop colostomy, suprapubic catheter placement, multiple orthopaedic repairs, and abdominal wall grafting. She was referred subsequently to our institution for consideration of pelvic reconstruction.

The patient was able to urinate via her urethra; however, she had a persistent vesicocutaneous fistula at her prior suprapubic site and required urinary tract infection prophylaxis due to recurrent infections.

A contrast enema of the proximal and distal colostomy was performed, demonstrating no stricture or fistula. A magnetic resonance imaging of the pelvis demonstrated severe atrophy and distortion of the pelvic floor muscles, left greater than right, and rightward-inferior deviation of the urethra.

**Fig. 2:**
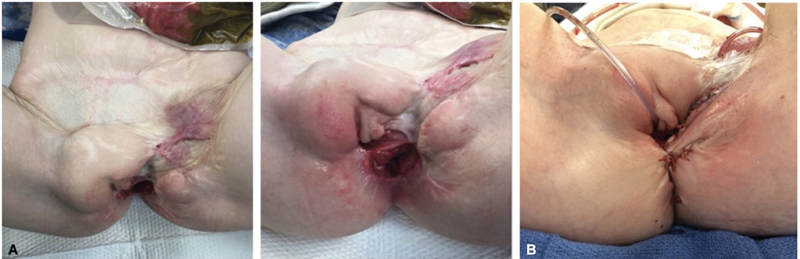
( **A** ) Exam under anesthesia findings with complete disruption of the perineal body and rightward displacement of the mons pubis. ( **B** ) Findings post-perineal sphincter reconstruction, introitoplasty, anoplasty, reconstruction of the perineal body, closure of the vesicocutaneous fistula, flap elevation of the mons pubis, and closure of the mucous fistula.

We performed an EUA which showed the posterior anus to be in proper position; however, the anterior anus was splayed open with complete disruption of the perineal body. The patient had good sphincter contractions centered to the right of the anus. The mons pubis was completely displaced to the patient's right, as well as the vagina, which was noted to have an interceding gap of scar tissue; the lower edge of the labia was displaced down to the level of the left thigh, and the clitoris was normal appearing ( Fig. 2A ).

On cystourethroscopy, the urethral meatus was in an orthotopic position despite deviation in the patient's pelvic anatomy and was easily entered with the cystoscope. There was normal coaptation of the bladder neck and the bladder appeared smooth-walled with orthotopic ureteral orifices. There was no evidence of vesicovaginal or urethrovaginal fistula. The vesicocutaneous fistula was identified and began to leak when the bladder was filled to 200 mL. There was no stress incontinence appreciated on abdominal palpation. A urodynamic study was performed, which showed diminished functional bladder capacity, detrusor overactivity, elevated voiding pressure, intermittent urine dribbling, detrusor sphincter dyssynergia, and incomplete bladder emptying. Vaginoscopy was remarkable for an obstructed vaginal introitus; however, the vaginal sidewalls were well visualized with normal-appearing mucosa.

The patient underwent reconstruction with the colorectal, urology, gynecology, and plastic surgery teams for perineal sphincter reconstruction, introitoplasty, anoplasty, reconstruction of the perineal body, closure of the vesicocutaneous fistula, flap elevation of the mons pubis, and closure of the mucous fistula. The procedure began with mobilization and elevation of the right mons pubis and the left labia by plastic surgery, followed by closure of the vesicocutaneous fistula by urology. The anterior border of the anus was mobilized and separated from the posterior wall of the vagina. Reconstruction of the perineal body was performed by tacking the sphincter muscles to the anterior rectal wall, and anoplasty was performed by suturing the anterior rectal wall to the anal skin. The posterior aspect of the anus was left untouched as it was in the proper position. An introitoplasty was completed by rotating the right vaginal tissue toward the front to provide a good vaginal lumen and this was sutured to the skin. The mons flap was rotated and sutured to the skin, over a drain ( Fig. 2B ).

At 2 weeks, a cystogram showed successful closure of the vesicocutaneous fistula without bladder extravasation; the patient was able to void spontaneously per her urethra. The anus was well-healed without stricture; the reconstructed perineal body was intact, and the surrounding sphincter complex was palpable on digital exam. On vaginoscopy, the patient was noted to have narrowing at the skin level of the introitus; however, the vaginal canal had healthy-appearing mucosa, and the cervix was well-visualized without abnormality. Cystoscopy also confirmed a well-healed repair. At this point, the colostomy was closed with creation of a Malone appendicostomy.

At follow-up 2 years after reconstruction, the patient was doing well and had complete bowel and urinary control and was no longer performing antegrade flushes.

## Discussion

We present three cases of extensive perineal injury from trauma, which led to the disruption of the anal sphincter complex and required reconstruction using the PSARP approach.

Perineal injury is rare in the pediatric population, especially to the extent observed in our cases, making surgical management particularly challenging due to the lack of a standardized therapeutic approach. These injuries can affect various pelvic structures, including the vulva, vagina, penis, testis, anus, rectum, urethra, and bladder, underscoring the need for a multidisciplinary approach to patient care.

Preparing for reconstructive surgery in cases of extensive perineal trauma requires a comprehensive assessment of the injuries. 4 5 This evaluation must be based on the mechanism of injury, visible injury patterns, and presenting symptoms. Preoperative evaluations including colostogram, vaginoscopy, and cystourethroscopy help to delineate the extent of the injuries and refine the surgical strategy.

Current management strategies for pediatric patients with perineal trauma are not well standardized and often vary based on injury severity and institutional practices. These approaches are frequently tailored to the preferences of the pediatric surgical team and are influenced by protocols and studies primarily derived from adult populations. In cases with isolated genital injuries, it is largely understood that primary repair can be successful. However, the optimal management strategy for injuries with genital and anorectal involvement remains unclear.

Usually, a diverting colostomy is performed at the initial injury to allow the perineal area to heal without the passage of stool. Some authors advocate for primary repair of the perineum even in the presence of extensive injuries, arguing that it can be both safe and effective depending on the injury's extent and the patient's overall condition. 1 3 5 9 10 11

The PSARP used first to repair infants born with an anorectal malformation enhanced functional outcomes for patients with anorectal malformations. 12 Over time, this technique has been adapted for other uses, including the repair of perineal injuries resulting from trauma and sexual assault. 2 5 13 The experience gained from using the posterior sagittal approach for reoperations in patients with anorectal malformations can be applied to reconstructions related to trauma. 14 A common theme in females is the need for reconstruction of the perineal body. 6 15 In males, mobilizing the sphincter to surround the anus and providing access to a retracted rectum were key aspects. 16 In our study, we applied the principles of the PSARP to the anal sphincter complex reconstruction with favorable results. Two of the patients were successfully managed with colostomy reversal and Malone appendicostomy as a bridge to continence; both have since discontinued their Malone flushes and have achieved full stool continence. One patient has his colostomy reversal and Malone appendicostomy planned, but on exam has developed concentric sphincter contraction, so we anticipate he will have bowel control.

## Conclusion

Pediatric pelvic trauma is rare. It can cause devastating disruptions to physiology, particularly when the anal sphincter is involved. These cases are difficult to treat; however, experience from treating re-operative anorectal malformation cases can be utilized. Through each of our cases, in collaboration with colorectal surgery, urology, gynecology, orthopaedics, and plastic surgery, we show that rectal repair and anal sphincter reconstruction via a posterior sagittal approach can result in restoration of fecal continence with favorable cosmetic outcomes.

## Notes

Importance for a Pediatric Surgeon Pediatric pelvic trauma can cause devastating disruptions of physiology and are difficult to treat. Experience from reoperations for anorectal malformations can be applied to these cases, including the use of a multidisciplinary team and posterior sagittal approach.
